# Clinical Impact of the CTLA4 rs231775 Polymorphism in Acute Myeloid Leukemia Treated with Autologous Stem Cell Transplantation

**DOI:** 10.3390/cancers18111734

**Published:** 2026-05-26

**Authors:** Elisa Tarozzi, Katja Seipel, Inna Shaforostova, Marie-Noelle Kronig, Ulrike Bacher, Thomas Pabst

**Affiliations:** 1 Department of Medical Oncology, University Hospital Bern, 3010 Bern, Switzerland; elisa.tarozzi@students.unibe.ch (E.T.); innaivanovna.shaforostova@insel.ch (I.S.); marie-noelle.kronig@insel.ch (M.-N.K.); 2 Department for Biomedical Research (DBMR), Bern University, 3008 Bern, Switzerland; 3 Department of Hematology, University Hospital Bern, 3010 Bern, Switzerland; veraulrike.bacher@insel.ch

**Keywords:** acute myeloid leukemia (AML), autologous stem cell transplantation (ASCT), allogeneic transplantation (allo-HSCT), antileukemic immune surveillance, progression-free survival (PFS), overall survival (OS), cytotoxic T-lymphocyte-associated protein 4 (CTLA4), single-nucleotide polymorphism (SNP)

## Abstract

Background: Germline variants of the immune checkpoint receptor *CTLA4* may modulate T-cell activation, potentially influencing post-transplant immune reconstitution and clinical outcomes. Methods: In this retrospective single-center study, 140 AML patients who underwent ASCT were stratified into three groups according to the *CTLA4* rs231775 geno-types A17hom, T17Ahet, and T17hom. Clinical outcomes, including overall survival (OS) and progression-free survival (PFS), were evaluated. Multivariate analysis was performed to adjust for known prognostic covariates. Results: Baseline clinical characteristics varied according to *CTLA4* genotype with a higher proportion of favorable cytogenetic risk in A17hom carriers. Comparative analysis revealed differences in survival rates, with superior outcomes in A17hom carriers. In multivariate analysis directional trends persisted across all endpoints. Conclusion: The prognostic impact of *CTLA4* rs231775 on post-ASCT outcomes suggests that T-cell inhibitory signaling may contribute to anti-leukemic immune surveillance in the autologous setting. These findings provide a rationale for investigating *CTLA4* inhibition as consolidation therapy following ASCT in future prospective studies, particularly in T17hom carriers, who may harbor a less favorable immune profile. *CTLA4* genotyping at diagnosis may represent a practical tool to support risk stratification in AML.

## Simple Summary

Inherited variants in immune checkpoint genes may affect leukemia susceptibility and clinical outcomes. In this study, we investigated the clinical impact of the common *CTLA4* polymorphism rs231775 in AML patients undergoing ASCT. The *CTLA4* A17hom germline variant was associated with superior survival following ASCT, suggesting this variant as a candidate prognostic biomarker in risk stratification and selection of personalized therapeutic strategies in AML.

## 1. Introduction

Acute myeloid leukemia (AML) is an aggressive hematologic malignancy characterized by the clonal expansion and differentiation arrest of immature myeloid progenitors, resulting in bone marrow failure and peripheral cytopenia [1,2]. In Europe, AML represents the most common myeloid malignancy in adults, with a median age at diagnosis of approximately 65–70 years [3,4]. The age-standardized incidence rate is estimated at 3.7–4.7 cases per 100,000 individuals per year, with a marked increase in the elderly population [3,4]. Despite its substantial incidence, prevalence remains low—estimated at approximately 11 cases per 100,000—reflecting the aggressive clinical course and high disease-related mortality associated with this condition [3].

AML is biologically and clinically heterogeneous, driven by a complex interplay of cytogenetic abnormalities, somatic mutations, epigenetic alterations, and microenvironmental factors that collectively contribute to leukemogenesis and therapeutic resistance [5,6,7]. Large-scale genomic analyses conducted across diverse AML cohorts have further highlighted the polygenic nature of this disease and the convergence of multiple dysregulated molecular pathways underlying leukemic transformation [8,9]. Improved characterization of this molecular complexity has substantially refined prognostic assessment and therapeutic stratification. The 2022 European LeukemiaNet (ELN) recommendations now integrate molecular and cytogenetic alterations into standardized risk categories that guide treatment decisions and transplantation strategies [10].

Despite advances in chemotherapeutic regimens, approximately 30% of patients fail to achieve complete remission and more than 50% will ultimately experience relapse [11,12]. Allogeneic hematopoietic stem cell transplantation (allo-HSCT) remains the most effective consolidation strategy for relapsed or high-risk AML [11]. However, treatment-related toxicity, graft-versus-host disease, and donor availability substantially limit its applicability. Autologous stem cell transplantation (ASCT) therefore remains an important post-remission strategy in selected patients, demonstrating improved relapse-free and overall survival compared with chemotherapy consolidation alone [13,14,15]. Nevertheless, significant inter-individual variability in post-ASCT outcomes persists, underscoring the need for refined prognostic tools capable of improving post-remission strategies.

In this context, the immune checkpoint receptor CTLA4 has emerged as a molecule of particular interest because of its central role as a negative regulator of T-cell activation and immune tolerance [16,17]. CTLA4-mediated immune modulation contributes to the graft-versus-leukemia (GvL) effect in the allogeneic setting [18,19] and more broadly influences antitumor immune surveillance [20]. In the autologous setting, the allogeneic immune effect is absent, and the therapeutic benefit of ASCT primarily relies on high-dose chemotherapy-induced cytoreduction [21,22]. Nevertheless, accumulating evidence indicates that ASCT is followed by profound immune reconstitution characterized by expansion and maturation of T-cell subsets, recovery of CD8^+^ cytotoxic T lymphocytes and natural killer cells, restoration of antigen presentation, and progressive diversification of the immune repertoire [22]. Similar immune resetting phenomena have also been described in autoimmune diseases treated with ASCT, where transplantation may promote the development of a newly self-tolerant immune system [21,23,24]. Together, these observations provide a biological rationale for investigating immune regulatory pathways, including CTLA4 signaling, in the autologous transplantation setting.

Inter-individual variability in CTLA-4 expression and function may be partly determined by germline genetic variation. Among the identified polymorphisms, rs231775 has attracted particular interest because of its association with multiple immune-mediated disorders and malignancies [25,26]. This single-nucleotide polymorphism consists of an A-to-G transition at position 49 of exon 1 of the *CTLA4* gene, resulting in a threonine-to-alanine amino acid change at codon 17 (Thr17Ala) [27]. Located within the signal peptide region, this variant has been shown to influence CTLA4 intracellular processing, trafficking, and surface expression, thereby modulating downstream T-cell inhibitory signaling. [28]. According to the Allele Frequency Aggregator (ALFA) project, the global minor allele frequency (MAF) of rs231775 is 0.37 [29], with an estimated prevalence of 14% for the Ala17 homozygous genotype, 46% for the Thr17Ala heterozygous genotype, and 40% for the Thr17 homozygous genotype [30]. While the clinical relevance of CTLA4 polymorphisms has been primarily investigated in the allogeneic transplantation setting, evidence regarding their role after ASCT remains scarce. Notably, a recent study examined the disease stage-dependent clinical impact of the rs231775 variant in multiple myeloma patients undergoing ASCT, demonstrating that the AA genotype was associated with inferior progression-free survival (PFS) in ISS stages I–II, but with superior PFS in ISS stage III, suggesting a context-dependent immunomodulatory role of this variant in the autologous setting [31].

We therefore sought to determine whether the CTLA4 rs231775 polymorphism influences overall survival (OS) and progression-free survival (PFS) in AML patients undergoing ASCT, with the aim of identifying a readily accessible germline biomarker capable of refining post-remission risk stratification.

## 2. Materials and Methods

### 2.1. Study Design and Patient Cohort

This single-center retrospective study enrolled 140 patients with acute myeloid leukemia (AML) treated at the Department of Medical Oncology, Bern University Hospital, Switzerland, between 2004 and 2025. All patients provided signed informed consent prior to inclusion. The study was approved by the local Ethics Committee of the Canton of Bern, Switzerland (decision number: 2025-00853; date of approval 11 June 2025).

Patients were risk-stratified according to the European LeukemiaNet (ELN) classification into favorable, intermediate, and adverse risk groups, based on cytogenetic findings and molecular mutations assessed at diagnosis. For historical reference, the French–American–British (FAB) classification subtype was also recorded; this morphology-based system, which defines AML subtypes (M0-M7) according to cytological and cytochemical criteria, has since been largely superseded by the World Health Organization (WHO) classification [32].

All patients were managed according to a standardized institutional treatment approach consisting of two cycles of induction chemotherapy based on the conventional “7 + 3” regimen, comprising cytarabine and an anthracycline. Patients who achieved complete remission subsequently underwent peripheral blood stem cell mobilization and apheresis in preparation for autologous stem cell transplantation (ASCT), which was performed at Bern University Hospital. Prior to ASCT, patients received consolidation with a myeloablative, busulfan-based high-dose conditioning regimen. In the event of post-remission relapse, the indication for allogeneic stem cell transplantation (allo-HSCT) was evaluated on an individual basis within the institutional multidisciplinary tumor board.

ASCT is considered the standard of care at our center for patients with favorable or intermediate-risk AML, in line with HOVON protocols. In selected cases, such as the absence of a suitable donor or patient refusal of allo-SCT, ASCT was also performed in patients with adverse-risk AML. This approach allowed the evaluation of post-transplant outcomes across different risk categories. Acute promyelocytic leukemia (APL, M3 subtype) was excluded, as autologous transplantation is not generally recommended in this subgroup [33]. Notably, one patient with APL underwent ASCT after achieving complete remission.

### 2.2. CTLA4 Gene Analysis

Genomic DNA was extracted from peripheral blood mononuclear cells (PBMCs). A fragment encompassing exon 1 of the CTLA4 gene was amplified by PCR using FIREPol DNA polymerase (Solis BioDyne, Tartu, Estonia) and gene-specific primers (forward: 5′-CTGAAGACCTGAACACCGCTCCCA-3′; reverse: 5′-CACCTCCTCCATCTTCATGCTCCA-3′). PCR products were subjected to Sanger sequencing (Microsynth AG, Balgach, Switzerland). Genotype frequencies were tested on a Hardy–Weinberg equilibrium calculator (HWE) [34]. The observed allele frequencies in our cohort were subsequently compared with those reported for the European population in the Allele Frequency Aggregator (ALFA) project [29].

### 2.3. Assessment and Endpoint

Treatment response was assessed according to the European LeukemiaNet (ELN) recommendations [10]. The primary endpoints of this study were progression-free survival (PFS) and overall survival (OS). PFS was defined as the time in months from autologous stem cell transplantation (ASCT) to the first documented event—disease relapse, death from any cause, or last follow-up—whichever occurred first. OS was defined as the time in months from ASCT to death from any cause or last follow-up. Patients lost to follow-up were censored at the date of their last known contact. Patients who subsequently underwent allogeneic hematopoietic stem cell transplantation (allo-HSCT) following relapse after ASCT were censored at the time of allo-HSCT, given that this salvage procedure represents a subsequent line of therapy that could independently influence long-term outcomes. Data collection was completed in December 2025.

### 2.4. Statistical Analysis

The *CTLA4* polymorphism rs231775 was stratified into three genotypes: homozygous carriers of the major allele (AA; T17hom), homozygous carriers of the minor allele (GG; A17hom), and heterozygous carriers (AG; T17A het). Clinical data were collected from the EPIC electronic health record system at Bern University Hospital; complementary data on cell therapy procedures were retrieved from the Marcell software Version 8.3.3.0. Categorical variables were compared using the Chi-square test or Fisher’s exact test, as appropriate. Continuous non-parametric variables were compared across groups using the Kruskal–Wallis test, with pairwise comparisons performed using the Mann–Whitney U test. Progression-free survival (PFS) and overall survival (OS) were estimated using the Kaplan–Meier method and compared between groups using the log-rank test; the Wilcoxon test was additionally applied to assess differences in early survival. Univariate and multivariable analyses were performed using the Cox proportional hazards model to identify independent prognostic factors for PFS and OS. All statistical analyses were conducted using GraphPad Prism (version 11.0.0; GraphPad Software, San Diego, CA, USA) and RStudio (version 2025.05.1 + 513; Posit PBC, Boston, MA, USA) for Cox regression analyses. A two-sided *p*-value of ≤0.05 was considered statistically significant.

## 3. Results

### 3.1. Prevalence of the CTLA4 A17 Allele in European AML Patients

This study included 140 patients with acute myeloid leukemia (AML) in whom the *CTLA4* exon 1 sequence was determined. Of these, 62 patients (44%) were homozygous for the major allele encoding the CTLA4-T17 isoform (T17hom; AA genotype), 64 patients (46%) were heterozygous carriers of the rs231775 single nucleotide polymorphism (T17Ahet; AG genotype), and 14 patients (10%) were homozygous for the minor allele (A17hom; GG genotype). The minor allele frequency (MAF) observed in this cohort was 0.33, compared to MAF 0.36 in the European population by the Allele Frequency Aggregator project (www.ncbi.nlm.nih.gov/snp/docs/gsr/alfa/, accessed on 4 April 2026).

### 3.2. Baseline Clinical Characteristics of the AML Patient Cohort

Baseline clinical characteristics of the 140 patients with Acute Myeloid Leukemia were evaluated for the overall cohort as well as stratified according to the three CTLA4 rs231775 genotypes encoding either alanine or threonine at position 17 (CTLA4 A17hom, T17Ahet, and T17hom) (Table 1). In the entire cohort, the male-to-female distribution was 74:66 (ratio 1.1), whereas a higher male predominance was observed in the A17hom subgroup (9:5; ratio 1.8). The median age at diagnosis was 54 years and showed no relevant differences among the three genetic subgroups. Most patients were diagnosed with de novo AML, while 14 patients had an alternative etiology, including eight cases of secondary AML arising from Myelodysplastic Syndromes (MDS) or Blastic Plasmacytoid Dendritic Cell Neoplasm (BPDCN) and six cases of therapy-related AML. Laboratory parameters at diagnosis, including hemoglobin levels, platelet count, white blood cell count, Lactate Dehydrogenase (LDH) levels, and blast percentages in peripheral blood and bone marrow were analyzed according to *CTLA4* genotype. The median levels of hemoglobin, LDH, and platelets were comparable in the three genetic subgroups indicating similar levels of anemia and thrombocytopenia. Leukopenia was more prevalent in the A17hom subgroup (*p* = 0.08), with more leukocytosis in the T17Ahet and T17hom subgroups (*p* = 0.55). Peripheral blast cell percentage was lower in the A17hom subgroup (8% vs. 50%, *p* = 0.08). Bone marrow blast percentage was lower in the A17hom subgroup (53% vs. 80%, *p* = 0.21). FAB classification varied according to *CTLA4* genotype with a prevalence of monocytic M4 in the A17hom subgroup (50% vs. 20%, *p* = 0.06). Cytogenetic aberrations and somatic mutations were present at balanced proportions in the three genetic subgroups. The ELN genetic risk classifications varied in the genetic subgroups with a higher proportion of favorable risk in the A17hom subgroup (79% vs. 44%, *p* = 0.006).

### 3.3. Treatment Outcomes-Univariate Analysis

**Figure 1:**
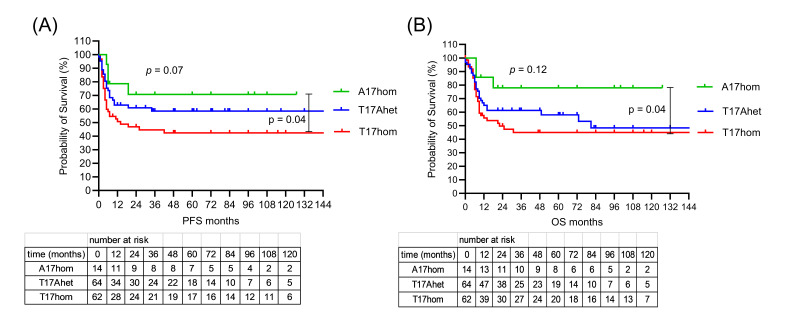
Survival outcomes in AML patients after ASCT according to *CTLA4* rs231775 genotype. (**A**) Progression-free survival (PFS) according to CTLA4 gene polymorphism rs231775 encoding CTLA4 A17hom, T17Ahet, or T17hom. (**B**) Overall survival (OS) in genetic subgroups CTLA4 A17hom, T17Ahet, or T17hom.

Post-ASCT clinical outcomes were evaluated in the overall cohort and the three genetic subgroups (Figure 1, Table 2). Progression-free survival (PFS) and overall survival (OS) differed according to CTLA4 rs231775 genotype, with higher survival rates observed in the A17hom, intermediate outcomes in the T17Ahet, and lower survival rates in the T17hom subgroup (Figure 1A,B). To assess potential differences in early events, PFS was analyzed in two statistical tests, Mantel–Cox (all events equal weight) and Wilcoxon (early events more weight). In pairwise comparisons, PFS was longer in the A17hom subgroup compared with T17hom (*p* = 0.057, Mantel–Cox test; *p* = 0.038, Wilcoxon test), and in the T17Ahet subgroup compared with T17hom (*p* = 0.09 for both tests). A statistically significant difference in OS was observed between A17hom and T17hom (*p* = 0.04 for both log-rank and Wilcoxon tests), whereas no significant difference was detected between T17Ahet and T17hom.

The overall response rate after ASCT was 93%, with 130 patients in complete remission (Table 2). Four-year PFS rates varied according to *CTLA4* genotype with 71% in A17hom, 58% in T17Ahet, and 42% in T17hom (*p* = 0.06). Four-year overall survival (OS) rates were 78%, 61%, and 45%, respectively (*p* = 0.04). The cumulative incidence of relapse was 29%, 38%, and 53%, respectively (*p* = 0.11), while mortality was 21%, 41%, and 50%, respectively (*p* = 0.13).

To assess the impact of single variables on survival hazard ratios were calculated in univariate Cox regression analysis. Both the GG (A17hom) and AG (T17Ahet) genotypes were associated with longer PFS and OS compared with the AA genotype (T17hom) (Table 3). For PFS, hazard ratios were 0.38 (95% CI, 0.14–1.08; *p* = 0.07) for A17hom and 0.63 (95% CI, 0.37–1.01; *p* = 0.08) for T17Ahet. For OS, the hazard ratio was 0.32 (95% CI, 0.10–1.05; *p* = 0.06) for A17hom, while no statistically significant association was observed for T17Ahet. Adverse ELN cytogenetic risk was significantly associated with shorter PFS (HR 2.08, 95% CI, 1.16–3.71; *p* = 0.01) and OS (HR 3.30, 95% CI, 1.86–5.88; *p* < 0.0001) compared with favorable risk. A peripheral blood blast count ≥50% at diagnosis was associated with shorter PFS (HR 1.79, 95% CI, 1.04–3.07; *p* = 0.03) and OS (HR 1.67, 95% CI, 0.96–2.91; *p* = 0.07). These variables were subsequently included in the multivariable analysis to assess their independent prognostic impact.

### 3.4. Treatment Outcomes—Multivariate Analysis

To identify independent prognostic factors, hazard ratios were calculated in multivariate analysis (Table 4). Adverse ELN risk at diagnosis was independently associated with significantly inferior progression-free survival (PFS; HR 2.37, 95% CI: 1.22–4.63; *p* = 0.01) and overall survival (OS; HR 3.65, 95% CI: 1.86–7.17; *p* = 0.0002). There was a substantial reduction in the risk of disease progression and death in carriers of the rs2311775 AG genotype (T17Ahet) compared with AA carriers (PFS HR 0.57, 95% CI: 0.31–1.03; *p* = 0.06; OS HR 0.60, 95% CI: 0.33–1.10; *p* = 0.1). Similarly, carriers of the GG genotype (A17hom) had a lower risk of both progression and death compared with AA carriers (HR 0.5, *p* = 0.2). A peripheral blast count of ≥50% at diagnosis was significantly associated with a higher risk of disease progression (HR 1.89, 95% CI: 1.08–3.31; *p* = 0.03), with a similar trend observed for OS (HR 1.65, 95% CI: 0.94–2.90; *p* = 0.08). Overall, the multivariate PFS model demonstrated moderate discriminatory ability, with a concordance index (C-index) of 0.66 (SE: 0.035), and the likelihood ratio test confirmed the overall model significance (*p* = 0.01). Similarly, the OS model showed a C-index of 0.67 (SE: 0.038), with the likelihood ratio test indicating strong overall model fit (*p* = 0.002).

**Table 4:** Clinical outcomes, multivariate analysis.

Predictors	PFS		OS	
	HR (CI)	*p*-Value	HR (CI)	*p*-Value
rs231775, T17Ahet vs. T17hom	0.57 (0.31–1.03)	0.06	0.60 (0.33–1.10)	0.10
rs231775, A17hom vs. T17hom	0.49 (0.17–1.45)	0.20	0.52 (0.15–1.80)	0.31
ELN risk, intermediate vs. favorable	1.61 (0.80–3.25)	0.19	1.84 (0.87–3.89)	0.11
ELN risk, adverse vs. favorable	2.37 (1.22–4.63)	0.01	3.65 (1.86–7.17)	0.0002
Peripheral blast, >50% vs. <50%	1.89 (1.08–3.31)	0.03	1.65 (0.94–2.90)	0.08
Age at diagnosis, >65 vs. <65	0.44 (0.19–1.03)	0.06	1.12 (0.58–2.20)	0.73
Male vs. female	1.42 (0.79–2.53)	0.24	1.14 (0.64–2.03)	0.65

ELN: European LeukemiaNet; PB: Peripheral blast cells (PB).

## 4. Discussion

The *CTLA4* rs231775 polymorphism has previously been associated with susceptibility to autoimmune diseases and solid tumor risk [25,26]. In the present retrospective cohort of 140 AML patients, the minor allele frequency (MAF) was 0.33, slightly lower than the 0.36 reported for the European population in the ALFA database (*n* = 262,130; www.ncbi.nlm.nih.gov/snp/docs/gsr/alfa/, accessed 4 April 2026). Whether this modest difference reflects a true biological signal—such as a potential protective role of the GG genotype (A17hom) against leukemia development—remains entirely speculative in the absence of a healthy control group and given the limited sample size of the present cohort. This observation is therefore reported descriptively, and no causal inference can be drawn. Adequately powered case-control studies will be required to formally evaluate the role of rs231775 in AML susceptibility.

Beyond disease predisposition, our data suggest that rs231775 may have prognostic value for outcomes following ASCT. A trend toward significance was observed for four-year PFS (71%, 58%, and 42% for AA, AG, and GG genotypes, respectively; *p* = 0.06), while four-year OS differed significantly across the three subgroups (78%, 61%, and 45%, respectively; *p* = 0.04). Although these findings did not consistently reach statistical significance for all comparisons, they suggest a possible genotype-associated gradient in post-transplant outcomes.

Prior studies have linked the AA genotype (T17hom) with higher CTLA4 expression [35,36], potentially resulting in stronger inhibition of T-cell activation and reduced antitumor immune response, which may be consistent with the poorer post-transplant survival observed in these patients. Conversely, the GG genotype (A17hom) has been associated with reduced CTLA4 surface expression and enhanced T-cell activation, which may improve antitumor immune surveillance [20,28,37]. Heterozygous carriers (AG/T17Ahet) showed intermediate outcomes for both OS and PFS, consistent with a potential dose-dependent biological effect. However, given the observational nature of the study, these interpretations remain speculative and require confirmation in mechanistic and prospective studies.

Kaplan–Meier analysis further supported these trends: pairwise comparison between A17hom (GG) and T17hom (AA) yielded *p* = 0.057 with the log-rank and *p* = 0.038 with the Wilcoxon test for PFS, and *p* = 0.04 by both tests for OS. The preferential weighting of early time points by Wilcoxon (Breslow) test for PFS suggests that the survival advantage associated with the A17hom genotype is most pronounced in the early post-transplant period. This finding is consistent with an immune-mediated mechanism acting during the phase of initial engraftment and immune reconstitution [38]. At longer follow-up, survival curves appeared to converge, indicating that the magnitude of any genotype-associated effect may diminish over time. These observations underscore the potential importance of administering consolidation therapy shortly after stem cell infusion to sustain early clinical benefit.

Regarding baseline characteristics, A17hom carriers displayed a significantly higher proportion of favorable cytogenetic risk (*p* = 0.006) and a trend toward lower peripheral blast counts at diagnosis (*p* = 0.08), suggesting that the rs231775 may influence both intrinsic disease biology and post-transplant immune responses. Multivariate analysis confirmed that adverse ELN cytogenetic risk was the dominant independent predictor of both inferior PFS and OS, with elevated peripheral blast count at diagnosis additionally predicting worse PFS but not reaching statistical significance for OS. After adjusting for these covariates, the prognostic effect of the rs231775 genotype was attenuated: T17Ahet carriers retained only borderline prognostic significance for both PFS and OS, while A17hom carriers showed a consistent but non-significant trend toward better outcomes. This attenuation is consistent with partial mediation of the genotype effect through cytogenetic risk, suggesting that rs231775 may act upstream by influencing the cytogenetic landscape of AML. Nevertheless, the persistence of directional trends across all endpoints, combined with the biological plausibility of CTLA4-mediated immune modulation following ASCT, indicates that an independent immunological contribution cannot be excluded. The limited sample size likely reduced statistical power, underscoring the need for validation in larger prospective cohorts.

Overall, our data support a model in which carrying at least one G allele, and particularly the homozygous GG genotype, provides a more favorable immunological and biological context in AML, both at diagnosis and following ASCT. The functional relevance of this variant is supported by the evidence that the threonine-to-alanine substitution encoded by rs231775 impairs glycosylation of the CTLA4 signal peptide during endoplasmic reticulum processing, resulting in reduced surface expression [30], thereby lowering the inhibitory threshold for T-cell activation. The adverse prognostic role of the AA genotype identified in our cohort is consistent with emerging evidence in other hematologic malignancies treated with ASCT, including multiple myeloma, where this genotype has similarly been associated with inferior outcomes in a subset of patients [31], further supporting rs231775 as a clinically relevant genetic determinant across different disease contexts.

The role of CTLA4-mediated immune regulation in AML is further supported by evidence from the allogeneic setting, where CTLA4 blockade with ipilimumab enhances the graft-versus-leukemia effect, with complete responses documented in relapsed AML patients in a prospective trial [39], and where the development of GvHD, reflecting potent donor T-cell activation, correlates with reduced relapse risk [40]. However, allogeneic HSCT is associated with substantial treatment-related mortality, significant GvHD morbidity, and the need for a compatible donor [41]. ASCT, by contrast, has markedly lower toxicity and is applicable to a broader patient population, but lacks the allogeneic immune pressure that underpins the GvL effect, which accounts for its higher relapse rates relative to allo-HSCT [42].

Collectively, the present data suggest that CTLA4-mediated immune regulation may contribute to inter-individual differences in post-ASCT outcomes in AML. The more favorable survival observed in A17hom and T17Ahet carriers raise the hypothesis that constitutively disinhibited autologous T cells may partially recapitulate a GvL-like effect in the autologous setting, a phenomenon that could be conceptualized as an “autologous graft-versus-leukemia reaction”. This is biologically plausible, as endogenous tumor-reactive T cells have been documented after ASCT, persist long-term, and correlate with clinical responses [43]; their functional expansion may be selectively favored in individuals carrying the hypomorphic rs231775 variant.

Although CTLA4 blockade has demonstrated clinical activity in relapsed AML following allogeneic HSCT [39], immune checkpoint inhibition has not yet been systematically investigated as post-ASCT consolidation therapy in AML. Preclinical studies using CRISPR-Cas9-mediated CTLA4 deletion have shown enhanced T-cell proliferation and reduced tumor burden in experimental leukemia models, providing mechanistic proof for therapeutic T-cell disinhibition in AML [20].

Taken together, these data suggest that combining ASCT with CTLA4 blockade may represent a clinically attractive strategy. If validated prospectively, the present findings may provide a strong biological rationale for such trials, particularly in T17hom patients, who may harbor constitutively higher CTLA4-mediated T-cell suppression and therefore derive the greatest benefit from pharmacological T-cell disinhibition. Genotyping rs231775 at diagnosis could guide post-transplant treatment stratification in AML, representing a simple and cost-effective tool to identify patients most likely to benefit from immunological consolidation strategies.

## 5. Conclusions

In conclusion, the *CTLA4* rs231775 polymorphism associated with post-ASCT outcomes in AML, with the A17hom genotype conferring superior PFS and OS and heterozygous carriers showing intermediate outcomes, is consistent with a dose-dependent effect. These findings suggest that rs231775 influences AML biology both upstream—in association with favorable cytogenetic risk at diagnosis—and downstream, by modulating post-transplant immune responses. While the prognostic effect was partially attenuated after covariate adjustment, directional trends persisted across all endpoints, supporting a residual independent immunological contribution. The rs231775 polymorphism may serve as a candidate biomarker for post-transplant risk stratification. Prospective validation in larger cohorts and clinical trials evaluating CTLA4 checkpoint blockade as consolidation therapy post-ASCT in AML are warranted.

## Figures and Tables

**Table 1:** Patient characteristics at initial diagnosis.

Parameter	T17hom (*n* = 62)	T17Ahet (*n* = 64)	A17hom (*n* = 14)	All Patients (*n* = 140)	*p*-Value
Male, *n* (%)	29 (47)	36 (56)	9 (64)	74 (53)	0.43 ^1^
m/f ratio	0.9	1.3	1.8	1.1	
Age, median (range)	54 (22–72)	53 (17–74)	52 (24–70)	54 (17–74)	0.43 ^1^
Hemoglobin g/L, median (range)	88 (47–137)	90 (7–134)	91 (53–126)	90 (7–137)	0.84 ^1^
Leukocytes G/L, median (range)	11 (0.2–272)	14 (0.5–267)	5 (0.4–202)	12 (0.2–272)	0.58 ^1^
Leukopenia (LC < 4 G/L), *n* (%)	18 (29)	11 (17)	6 (43)	35 (25)	0.08 ^2^
Leukocytosis (LC > 10 G/L), *n* (%)	32 (52)	33 (52)	5 (36)	70 (50)	0.55 ^2^
Platelets G/L, median (range)	79 (5–714)	81 (4–229)	112 (11–187)	82 (4–714)	0.35 ^1^
LDH U/L, median (range)	733 (198–2538)	730 (116–3074)	822 (412–7108)	762 (116–7108)	0.46 ^1^
Blasts PB %, median (range)	49 (0–99)	56 (4–99)	8 (2–95)	48 (0–99)	0.08 ^1^
Blasts BM %, median (range)	80 (10–95)	85 (0–100)	53 (25–95)	80 (0–100)	0.21 ^1^
FAB classification, *n* (%)					0.32 ^3^
M0	5 (8)	4 (6)	0	9 (6)	
M1 *	17 (27)	19 (30)	2 (14)	38 (27)	0.46
M2	22 (35)	18 (28)	4 (29)	44 (31)	0.17
M3	0	1 (2)	0	1 (<1)	
M4	12 (19)	13 (20)	7 (50)	32 (23)	0.06
M5	5 (8)	6 (9)	2 (14)	13 (10)	0.76
M6	0	1 (2)	0	1 (<1)	
Pathogenesis					0.51 ^3^
De novo AML, *n* (%)	55 (89)	59 (92)	12(86)	126 (90)	
sAML (MDS/MPN-related), *n* (%)	4 (6)	2 (3)	2 (14)	8 (6)	
tAML (therapy-related), *n* (%)	3 (5)	3 (5)	0	6 (4)	
Cytogenetic aberrations, *n* (%)					0.75 ^3^
Normal karyotype	42 (68)	39 (61)	8 (57)	89 (64)	
Complex karyotype	2 (3)	2 (3)	1 (7)	5 (4)	
Abnormal karyotype	17 (30)	17 (26)	4 (27)	38 (25)	
Somatic mutations, *n* (%)					0.24 ^3^
normal	16 (20)	11 (11)	5 (31)	32 (16)	
NPM1_mut_, FLT3_wt_	14 (18)	24 (24)	5 (31)	43 (22)	
NPM1_wt_, FLT3-ITD	5 (6)	1 (1)	0	6 (3)	
NPM1_mut_, FLT3-ITD	10 (13)	10 (10)	1 (6)	21 (11)	
Adverse risk genes	9 (11)	16 (16)	1 (6)	25 (13)	
Intermediate risk genes	19 (24)	28 (28)	2 (13)	49 (25)	
ELN-risk classification (2022), *n* (%)					0.006 ^2^
Favorable	27 (44)	36 (56)	11 (79)	74 (53)	
Intermediate	21 (34)	7 (11)	2 (14)	30 (21)	
Adverse	14 (23)	21 (33)	1 (7)	36 (26)	

BM: bone marrow; dm: double mutated; ELN: European LeukemiaNet; FAB: French–American–British; LDH: lactate dehydrogenase; mut: mutated; PB: peripheral blood; wt: wildtype; * includes: AML-M1, AML not further classifiable, AML from MDS (WHO 2008), and AML from MPN (WHO 2008). Abnormal karyotypes include t(8;21)(q22; q22), inv(16)(p13q22)/t(16;16)(p13;q22)., t(9;11)(p21;q23), inv(3)(q21q26)/t(3;3)(q21;q26), t(8;16)(p11;p13), t(6;11)(q27;q23), inv(7)(q22q36). Adverse risk genes include TP53, ASXL1, BCOR, EZH2, RUNX1, SF3B1, SRSF2, STAG2, U2AF1, ZRSR2; intermediate risk genes include DNMT3A, TETE2, NRAS, KRAS, IDH1, IDH2. ^1^ Kruskal–Wallis test. ^2^ Fisher’s exact test. ^3^ Chi-square test.

**Table 2:** Clinical outcomes and survival according to *CTLA4* genotype.

Outcomes and Survival	T17hom (*n* = 62)	T17Ahet (*n* = 64)	A17hom (*n* = 14)	All Patients (*n* = 140)	*p*-Value
Best response					0.44 ^1^
CR, *n* (%)	56 (90)	60 (94)	14 (100)	130 (93)	
No CR, *n* (%)	5 (8)	3 (5)	0	8 (6)	
Relapse, *n* (%)	33 (53)	24 (38)	4 (29)	61 (44)	0.11 ^1^
One-year PFS, *n* (%)	32 (51)	40 (63)	11 (79)	83 (59)	0.10 ^1^
Four-year PFS, *n* (%)	26 (42)	37 (58)	10 (71)	49 (35)	0.06 ^1^
Death, *n* (%)	31 (50)	26 (41)	3 (21)	60 (43)	0.13 ^1^
One-year OS, *n* (%)	34 (55)	42 (65)	12 (86)	88 (63)	0.08 ^1^
Four-year OS, *n* (%)	28 (45)	39 (61)	11 (78)	47 (34)	0.04 ^1^

CR: complete remission; PR: partial response; SD: stable disease; PD: progressive disease. OS: overall survival; PFS: progression-free survival. ^1^ Chi-square exact test.

**Table 3:** Clinical outcomes, hazard ratios, univariate analysis.

Predictors	PFS		OS	
	HR (CI)	*p*-Value	HR (CI)	*p*-Value
rs231775, A17hom vs. T17hom	0.38 (0.14–1.08)	0.07	0.32 (0.1–1.05)	0.06
rs231775, T17Ahet vs. T17hom	0.63 (0.37–1.01)	0.08	0.77 (0.46–1.30)	0.33
ELN risk, intermediate vs. favorable	1.53 (0.80–2.93)	0.19	1.83 (0.92–3.64)	0.08
ELN risk, adverse vs. favorable	2.08 (1.16–3.71)	0.01	3.30 (1.86–5.88)	0.0001
Peripheral Blast, >50% vs. <50%	1.79 (1.04–3.07)	0.03	1.67 (0.96–2.91)	0.07
Age at diagnosis, >65 vs. <65	0.62 (0.30- 1.30)	0.2	1.38 (0.77–2.48)	0.28
Male vs. female	1.40 (0.84–2.32)	0.2	1.10 (0.63–1.80)	0.83

ELN: European LeukemiaNet; PB: Peripheral blast cells (PB).

## Data Availability

Data available on request due to restrictions, privacy and ethics.
